# Unlocking the Therapeutic Potential: Selenium and Myo-Inositol Supplementation in Thyroid Disorders—Efficacy and Future Directions

**DOI:** 10.3390/life15101500

**Published:** 2025-09-24

**Authors:** Chinnu George Samuel, Parul Singh, Hala Abdullahi, Ibrahim Ibrahim

**Affiliations:** 1 Clinical Trial Office, Sidra Medicine, Doha P.O. Box 26999, Qatar; cgeorgesamuel@sidra.org; 2 Research Department, Sidra Medicine, Doha P.O. Box 26999, Qatar; psingh@sidra.org; 3 Sidra Medicine, Weill Cornell Medical College-Qatar, Doha P.O. Box 24144, Qatar; habdullahi@sidra.org

**Keywords:** thyroid diseases, selenium, myo-inositol, Hashimoto thyroiditis, Graves’ disease

## Abstract

**Background/Objectives**: Thyroid disorders such as Hashimoto’s thyroiditis and Graves’ disease represent major endocrine challenges worldwide, often requiring long-term management. Recently, nutritional supplementation with selenium and myo-inositol has been proposed as a supportive strategy. This review aims to summarize the current evidence regarding their efficacy in improving thyroid function, reducing thyroid autoantibodies in Hashimoto’s disease, and restoring biochemical euthyroidism in Graves’ disease. **Methods**: A narrative review of the available literature was undertaken, concentrating on randomized controlled trials and observational studies evaluating selenium and myo-inositol, alone or in combination (MYO+Se), in patients with autoimmune thyroid disorders and benign thyroid nodules. **Search Strategy and Study Selection**: We searched MEDLINE/PubMed, Embase, Cochrane CENTRAL, and Scopus from inception to 31 July 2025. The search used Boolean operators to combine the following keywords: (“selenium” OR “selenomethionine”) AND (“myo-inositol” OR “inositol”) AND (thyroid OR Hashimoto* OR Graves’ OR hyperthyroid* OR hypothyroid* OR nodule* OR goiter OR orbitopathy). We included human studies in English. **Inclusion criteria**: Research designs include RCTs, quasi-experimental studies, cohort/case-control studies, and big case series (*n* ≥ 30). **Exclusion criteria**: Animal-only or in vitro studies (unless mechanistic), pediatric case reports, and editorials/commentaries. **Study selection and data extraction:** Two reviewers screened independently; discrepancies were settled through consensus. The data retrieved included the population, baseline iodine/selenium status (if reported), dose/formulation, treatment duration, outcomes (TSH, FT4, FT3, TPOAb, TgAb, TRAb, nodule metrics), and adverse events. **Quality assessment**: The risk of bias was assessed using the RoB-2 for RCTs and the Newcastle-Ottawa Scale or JBI checklists for observational studies. A qualitative synthesis emphasized study quality, consistency, directness, and accuracy. **Results**: Clinical research indicate that selenium supplementation may reduce thyroid peroxidase antibody (TPOAb) levels in Hashimoto’s disease, thereby attenuating autoimmune activity. Myo-inositol, particularly when combined with selenium, has been proven to improve thyroid hormone profiles while also lowering nodule size or growth. In Graves’ disease, supplementation has been linked to the restoration of biochemical euthyroidism in certain patients, albeit data are limited. Despite these encouraging results, diversity in trial design, treatment length, and dosages restrict the robustness of existing recommendations. **Conclusions**: Selenium and myo-inositol supplementation have shown promise as adjuvant treatments for autoimmune thyroid diseases and benign thyroid nodules. However, further large-scale, well-designed clinical trials are needed to determine the appropriate dosages, treatment duration, and patient selection criteria. Personalized supplementation solutions may improve medication efficacy and help with more comprehensive thyroid disease care.

## 1. Introduction

The human body’s iodine balance and the production and secretion of thyroid hormones (THs) are both controlled by the thyroid, an endocrine gland situated in the inferior, anterior neck [1]. Almost all vertebrates, including humans, depend on THs for growth and development [2]. THs synthesis and secretion are finely modulated by the hypothalamic–pituitary–thyroid (HPT) axis [2]. The hypothalamic–pituitary–thyroid (HPT) axis tightly regulates the production and secretion of thyroid hormones [2]. Triiodothyronine or active thyroid hormone is produced by the thyroid in 10% of cases, and thyroxine, T4, or inactive thyroid hormone, in 90% of cases. Regulation of thyroid hormones starts at the hypothalamus. Thyrotropin-releasing hormone (TRH) is released by the hypothalamus, and it stimulates the anterior pituitary’s thyrotropin cells to release thyroid-stimulating hormone (TSH). TSH, in turn, regulates the secretion of thyroid hormones from the thyroid gland [3].

Thyroid hormones are essential for the maintenance of metabolic regulation across the life span and are focused almost exclusively in the body’s tissues [4]. The body can adjust to both thyroid function and pathological difficulties, such as stress, growth, weight change, aging, and pregnancy, thanks to their close integration with another endocrine axis [4]. Additionally, there is growing evidence linking thyroid dysfunction to an increased risk of developing other diseases such as depression and anxiety disorders, obesity, metabolic syndrome, renal disease, and cardiovascular disease [4].

The thyroid gland’s ability to produce thyroid hormones is influenced by the availability of iodine, a trace element that is unevenly distributed on Earth and is necessary for the synthesis of thyroid hormones (T4) and (T3) [5]. Insufficient iodine causes TSH to rise and T4 and T3 levels to fall. This results in the production of hydrogen peroxide (H_2_O_2_), a crucial step in the synthesis of thyroxine. The levels of selenium-dependent proteins, particularly GPX1 and GPX3, gradually decline in cases of selenium deprivation.

Myo-inositol and selenium supplementation may enhance thyroid function, regulate autoimmune activity, and stimulate thyroid hormone production [6,7]. Selenium is an essential trace element that is present in high concentrations in the thyrid gland. It plays a crucial role in the production and metabolism of thyroid hormones [6]. A mild deficiency of selenium can lead to the development of autoimmune diseases, including Hashimoto’s thyroiditis [8]. Myo-inositol is a type of sugar alcohol that is involved in the proper action of various endocrine hormones, including insulin, gonadotropins, and thyroid-stimulating hormone (TSH) [9]. Inositol also plays a vital role in the structural lipids of cells, the development of cell shape (morphogenesis), regulation of cytoskeleton rearrangement, cell growth and division, ovulation, and gluconeogenesis [10,11]. The combined supplementation of selenomethionine and myo-inositol has also been investigated in thyroid diseases [12]. Combined supplementation can have a synergistic effect in improving thyroid function and reducing inflammation in autoimmune thyroid diseases [12]. This review aimed to thoroughly analyze the various benefits and risks associated with the use of selenium and myo-inositol supplements for the prevention and treatment of various thyroid disorders.

### 1.1. Thyroid Diseases

**Figure 1:**
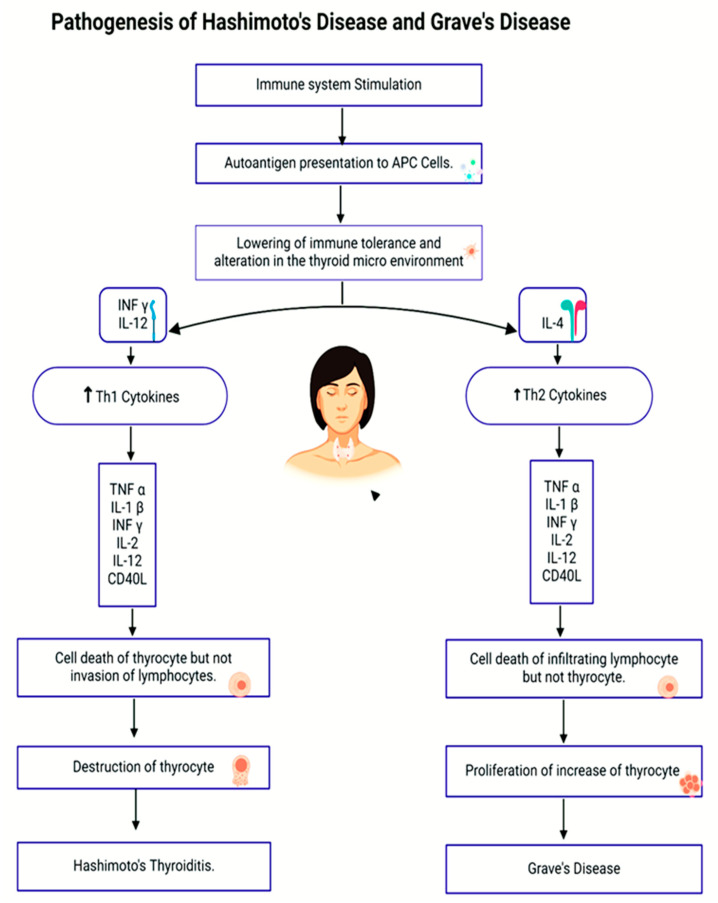
Pathogenesis of Hashimoto’s thyroiditis and Graves’ disease. In Hashimoto’s thyroiditis, anti-thyroid peroxidase (anti-TPO) and anti-thyroglobulin (anti-Tg) antibodies contribute to thyroid tissue destruction, leading to hypothyroidism. In Graves’ disease, thyroid-stimulating hormone receptor antibodies (TRAb) activate the TSH receptor, driving hyperthyroidism and orbitopathy, whereas blocking TRAb variants may result in hypothyroid features. Arrows indicates stimulatory pathways. Abbreviations: TNF, tumor necrosis factor; IL, interleukin; CD4, cluster of differentiation; IFN, interferon; Th1/Th2, T-helper subsets.

Thyroid diseases constitute a significant global health problem, affecting populations across diverse regions. Incidence has been reported in more than 110 countries, and approximately 1.6 billion individuals are considered at risk, underscoring their status as a recognized international public health concern [13]. The primary thyroid hormone-related disorders are outlined below; the pathophysiology of Graves’ disease and Hashimoto’s thyroiditis are summarized in Figure 1.

#### Autoantibodies and Clinical Associations in Autoimmune Thyroid Diseases

The pathophysiology and clinical presentation of autoimmune thyroid disorders are largely influenced by autoantibodies. Anti-thyroid peroxidase (anti-TPO) and anti-thyroglobulin (anti-Tg) antibodies are common in Hashimoto’s thyroiditis. Hypothyroidism, increasing thyroid tissue degradation, and an elevated risk of recurrent postpartum thyroiditis are all closely linked to these antibodies. Thyroid-stimulating hormone receptor antibodies (TRAb) are the main pathogenic factors in Graves’ disease. While inhibiting TRAb variations may contribute to hypothyroid phenotypes, stimulating TRAb binds to and activates the TSH receptor, resulting in hyperthyroidism and extrathyroidal characteristics such orbitopathy. Figure 1 depicts these connections by combining the antibody-mediated pathways and immune-cell interactions that underlie both disorders.

### 1.2. Hyperthyroidism

Elevated levels of thyroid hormone in the bloodstream, whether from endogenous or exogenous sources, stimulate hyperthyroidism [14]. The most frequent cause of hyperthyroidism is Graves’ disease-related scattered thyroid hyperplasia.

Graves’ disease (GD) is a syndrome characterized by ocular abnormalities, Graves’ orbitopathy (GO), a localized dermopathy known as pretibial myxedema (PTM), and an enlarged and hyperactive thyroid gland known as Graves’ hyperthyroidism [15]. When patients are affected by Graves’ hyperthyroidism, the TSHR-specific T cells and the presence of TSHR autoantibodies can result in extrathyroidal problems including PTM and GO.

Clinically substantial extrathyroidal complications might result from the interaction of autoreactive immune cells and TRAb with TSHR, which is also expressed in non-thyroidal tissues such fibroblasts and adipocytes. More thorough imaging indicates that moderate forms of GO are significantly more common in people with GD, even though GO is clinically evident in only about 5% of patients [16]. In a comparative study of hyperthyroidism patterns in low-iodine East Jutland, Denmark, and high-iodine Iceland, East Jutland shows more cases of nodular toxic goiter in the elderly, while Iceland sees a higher incidence of Graves’ disease in the young, implying that mild iodine deficiency leads to thyroid nodules in the elderly, and excessive iodine intake may induce Graves’ disease in the young [17]. A variant located within an intron of the HLA complex P5 (*HCP5*) gene, specifically the rs3094228 variant, has been found to have a separate association with both the susceptibility to GD and the age at which the disease begins in populations of Caucasian descent [18].

### 1.3. Hypothyroidism

Hypothyroidism means a low level of circulating thyroid hormones [19].

The commonest cause of hypothyroidism is an autoimmune thyroid disease known as Hashimoto’s thyroiditis (HT), also called chronic lymphocytic or autoimmune thyroiditis, which is characterized by increased thyroid volume, lymphocyte infiltration of the thyroid gland, and the presence of antibodies specific to thyroid antigens. HT, along with Graves’ Disease (GD), is considered one of the autoimmune thyroid disorders (AITDs) which have significantly increased in incidence in recent years [20].

A study conducted on a non-Caucasian Algerian population was the first to demonstrate the genotypic association between an intronic variant in the *IP6K3* gene and a reduced risk for HT. However, the study did not confirm the association between genetic variants in SDK2 and GNA14 with HT. IP6K3, located near the major histocompatibility complex, has previously been linked to other autoimmune diseases such as Graves’ disease and rheumatoid arthritis, indicating its potential role in the development of autoimmunity and HT [20].

## 2. Review

### 2.1. Role of Selenium and Myo-Inositol Supplementation in Thyroid Diseases

#### 2.1.1. Role of Selenium in the Thyroid

The microelement selenium (Se), which is necessary for life, is essential for maintaining the homeostasis of various vital processes, including immune–endocrine function and signaling transduction pathways [21]. Specifically, selenium (Se) is very prevalent in the thyroid gland and is essential for thyroid function. Nevertheless, epidemiological studies have shown that a significant portion of people worldwide suffer from Se insufficiency [22]. 

**Figure 2:**
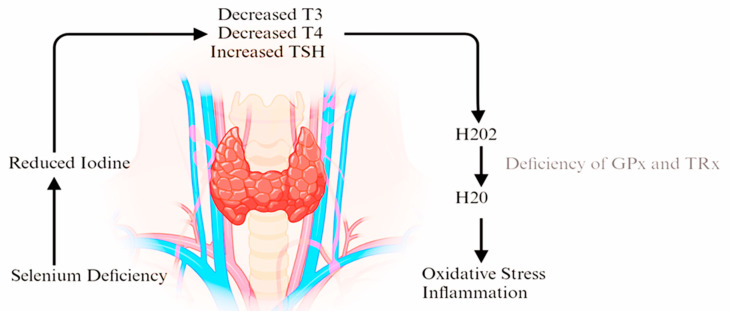
Effect of selenium deficiency in the thyroid gland. Selenium deficiency reduces the activity of selenoproteins such as glutathione peroxidases (GPx1/3), thioredoxin reductases (Trx), and deiodinases (DIOs). Reduced antioxidant capacity results in hydrogen peroxide (H_2_O_2_) accumulation, thyroid tissue oxidative stress, and progressive follicular cell damage. This can exacerbate autoimmune processes and impair T4-to-T3 conversion. Abbreviations: T3, triiodothyronine; T4, thyroxine; TSH, thyroid-stimulating hormone; GPx, glutathione peroxidase; Trx, thioredoxin reductase; H_2_O_2_, hydrogen peroxide.

Selenium was found to play a role in thyroid function and it is postulated that the link is through a specific enzyme called type-1 5′-deiodinase, which is involved in the conversion of thyroid hormones, which contains selenium [23]. This connection has also been confirmed in all three types of iodothyronine deiodinase enzymes [24]. Figure 2 summarizes the effect of Selenium deficiency in the thyroid gland.

In cases of selenium deficiency, the levels of selenoproteins, such as GPX1 and GPX3, decrease gradually. This results in the widespread distribution of H_2_O_2_ throughout the thyroid tissue, causing inflammation and ultimately leading to the destruction of the thyroid gland, as illustrated in Figure 2. The three main families of selenoproteins are glutathione peroxidases (GPxs) with seven genes, thioredoxin reductases (TRxs) with three genes, and iodothyronine deiodinases (DIs) with three genes. GPxs act as oxidoreductases and help protect cells from oxidative stress. TRxs form a redox system crucial for cell development and proliferation. DIs are responsible for converting thyroxine (T4) to triiodothyronine (T3) and play a role in the production of T3 in the body [25]. Selenium helps regulate the conversion of thyroid hormones by influencing the activity of enzymes called deiodinases. These enzymes are responsible for removing specific iodine atoms from thyroid hormones, which control the conversion of thyroxine (T4) to triiodothyronine (T3) and reverse T3 (rT3) [26].

Patients who have been newly or recently diagnosed with Graves’ disease typically exhibit low levels of selenium and produce thyroid-stimulating immunoglobulin, which leads to the overproduction of thyroid hormones [27]. Lane et al. conducted a study to investigate the serum selenium levels in patients with Graves’ disease who experienced remission and relapse, over 20 months [28]. The results of the study indicated that subjects who achieved remission had higher serum selenium levels of >120 μg/L, which was associated with a beneficial effect. Furthermore, the median values of TSH receptor autoantibodies (TRAb) were significantly reduced (*p* < 0.0001) in the remission group compared to the relapse group [28]. A systematic review and meta-analysis on the impact of selenium supplementation on Graves’ disease concluded that the supplementation of selenium aids in achieving biochemical euthyroid restoration [29]. Mantovani et al. also conducted a meta-analysis on the effect of selenium supplementation in Graves’ disease, which similarly yielded positive results in enhancing the restoration of biochemical euthyroidism [30]. However, the study suggests that further randomized controlled trials (RCTs) are needed in the future to obtain more conclusive results [30]. In contrast, a double-blinded, placebo-controlled trial on the daily supplementation of selenium in hyperthyroid patients with Graves’ disease showed no positive effects on recurrence rate [31]. Calissendorff et al. conducted an interventional study in Sweden involving 38 patients, where the intervention consisted of 200 μg/d of selenium versus a placebo. At 18 and 36 weeks following supplementation, the study discovered that the levels of free thyroxine (FT4) in the selenium group had dramatically dropped more than in the placebo group [14]. Furthermore, at 18 weeks, the selenium group experienced a much higher increase in levels [32].

In other observational studies, low selenium levels have been associated with a higher incidence of thyroid dysfunction [33]. Importantly, the evidence on Graves’ disease is still preliminary and limited. Larger, better-designed studies are necessary, as evidenced by the variable and underpowered results of some trials that indicate benefits for biochemical recovery or antibody decrease. The benefits of supplementing with selenium in cases of Hashimoto’s thyroiditis have been the subject of several investigations. In one study, individuals who received 200 µg/day of oral selenium for six months saw a substantial decrease in their titers of thyroglobulin antibody (TgAb) and thyroid peroxidase antibody (TPOAb) [34]. Additionally, a Cochrane review indicated that selenium supplementation may have potential benefits for patients with Hashimoto’s thyroiditis by reducing antibody levels and associated symptoms such as drowsiness, asthenia, and mood swings [35]. Research by Rostami et al. revealed that hypothyroid patients had lower selenium levels compared to controls [36]. Similarly, Erdal et al. demonstrated that serum selenium levels were lower in patients with Hashimoto’s thyroiditis (67.7 ± 10.4 mg/L) compared to the control group (83.7 ± 17.3 mg/L) [18].

Several studies have investigated the effects of selenium supplementation in patients with Hashimoto’s thyroiditis. A prospective randomized trial conducted in Greece found that 200 μg/day L-selenomethionine significantly reduced anti-TPO levels over 12 months of treatment (*p* < 0.0001) [37]. However, another prospective study in patients with subclinical hypothyroidism due to Hashimoto’s thyroiditis found no significant changes in TPOAb, CXCL9, CXCL10, or CXCL11 levels, but a decrease in TSH level was observed following 83 µg/day selenium supplementation for six months. L-selenomethionine supplementation at 83 µg/day during pregnancy and for six months after delivery was found to be safe and effective in lowering autoantibody titers and preventing postpartum relapse of thyroiditis in a randomized, double-blind, placebo-controlled study involving pregnant women with thyroiditis (*p* < 0.01).

Selenium supplementation in Hashimoto’s thyroiditis appears to enhance the activity of selenoproteins, leading to reduced local inflammatory reactions and a decrease in anti-TPO antibody production, ultimately improving the structure of the thyroid. Meanwhile, in Graves’ disease, selenium supplementation may support the restoration of euthyroidism and exert beneficial effects on the progression of mild to medium orbitopathy [29]. It has been shown that postpartum supplementation of selenium can reduce thyroid inflammatory activity and the incidence of hypothyroidism (*p* < 0.01), as well as decrease the risk of postpartum thyroiditis (PPT) [30]. Table 1 summarizes the evidence of the use of selenium in different thyroid disease etiologies and their results.

Notably, there is a great deal of variation in selenium supplementation protocols. Trial dosages have varied from 83 µg/day to 200 µg/day; some research indicates that greater doses (≥200 µg/day) result in more noticeable decreases in TSH levels and antibody titers [32,37], whereas lower doses (83–100 µg/day) have only mild or no effects. Furthermore, the length of treatment ranges from six to fifteen months, and different formulations (such as sodium selenite, L-selenomethionine, and selenium yeast) may have varying effects. These variations in dosage, time, and preparation probably account for the variable outcomes observed in different trials.

Overall, the current evidence is not conclusive enough to support the routine addition of selenium in the management of patients with Graves’ disease or Hashimoto’s thyroiditis. Therefore, future well-designed randomized placebo-contro4lled clinical trials in diverse ethnic backgrounds and different risk factor profiles are necessary to confirm the effects of selenium supplementation in patients with Hashimoto’s thyroiditis and provide evidence to support clinical decision-making.

#### 2.1.2. Role of Myo-Inositol and Selenium Combination in Thyroid

Myo-inositol is a molecule that plays an important role in both thyroid physiology and pathology. It functions as a second messenger that helps regulate intracellular calcium levels, which is critical for many cellular processes [40]. In 1850, a German physician named Johann Joseph Scherer first isolated inositol from muscle tissue, marking the beginning of its scientific discovery [10]. In a clinical study conducted by Nordio et al., the effectiveness of myo-Ins-Se, which includes myo-inositol at a dose of 600 mg/day and selenium at a dose of 83 µg/day, was examined in 86 patients with Hashimoto’s disease and subclinical hypothyroidism over a six-month period [41]. The results showed a significant improvement in thyroid function, as indicated by the decrease in TSH levels from 4.32 ± 0.06 mIU/L at baseline to 3.12 ± 0.09 mIU/L (*p* ≤ 0.001), as well as an improvement in the patient’s quality of life [41]. A dose of 4 to 30 g of myo-inositol per day is usually well tolerated; dosages greater than 12 g per day may cause minor gastrointestinal intolerance (e.g., nausea, diarrhea) [42]. A study conducted in 2018 investigated the safety and efficacy of MYO (600 mg/day) and Se (83 µg/day) supplementation in pregnant women from the first to third trimester and found that it normalized TSH, free T3, and free T4 levels [40]. Another prospective randomized double-blinded controlled study by Nordio M. involved women with autoimmune thyroiditis and TPO Ab levels above 350 IU/mL, TSH levels ranging from 4.01 to 9.99 mIU/L, and normal free T4 levels (0.6–1.8 ng/dL). The study aimed to restore TSH levels to less than 4 mIU/L. The results demonstrated that subclinical hypothyroid patients treated with selenomethionine showed positive effects, which were further enhanced by co-therapy with myo-inositol, likely due to the presence of autoantibodies (TPOAb and TgAb) [43].

TSH levels in subclinical hypothyroidism cohorts have decreased, indicating that myo-inositol may alter TSH signaling over the IP_3_/Ca^2+^ second-messenger pathway [44]. A study conducted on 642 patients with suspected hypothyroidism, who were treated with myo-inositol 600 mg/day plus selenium 83 µg/day for 6 months, reported a reduction in the size, number, and elasticity score of thyroid nodules. Additionally, the study showed a significant decrease in TSH levels, which dropped from 4.2 ± 0.21 mIU/L at baseline to 2.1 ± 0.20 mIU/L post-treatment [45]. Elevated TSH levels have been linked to thyroid cancer in some studies [34,35]. AKT, a signaling molecule of the protein kinase B family, is activated in many human cancers and is part of the phosphatidylinositol 3-kinase pathway. Normal thyroid expresses all three isoforms of AKT (AKT1, AKT2, and AKT3), and AKT plays a crucial role in the development and progression of thyroid cancer [46].

In a randomized controlled trial, 168 patients aged 22–62 years with TSH levels between 3–6 mIU/L, elevated TPO, and normal free T4 and T3 levels were enrolled for 6 months. They were randomized to receive either 83 µg/day of selenium or a combination of 600 mg/day of myo-inositol and 83 µg/day of selenium. The group that received the combination therapy had lower TSH levels, lower thyroglobulin antibody titers, and higher T3 and T4 levels in patients with Hashimoto’s disease. Both groups showed a reduction in TPO antibody concentration. The combination therapy also showed concomitant protection against cardiovascular complications [47]. Another study by G. Briguglia aimed to identify the efficacy of myo-inositol and selenium combination therapy for 6 months in subclinical hypothyroidism. It found reduced levels of TSH and autoantibodies and increased T3 and T4 levels. Remarkable enhancement in antibody titers and hormonal levels was observed within 3 months of therapy (*p* ≤ 0.05) [48]. A preliminary in vitro study demonstrated the potential benefits of myo-inositol, selenomethionine, or their combination on peripheral blood mononuclear cells (PBMCs) exposed to hydrogen peroxide (H_2_O_2_)-induced oxidative stress in women with Hashimoto’s thyroiditis (HT) as well as in the control group.

Heterogeneity in treatment duration and dosage is also noticeable across combination trials. Myo-inositol at 600–1200 mg/day and selenium at 83 µg/day for 3–12 months were examined in the majority of randomized trials; there is evidence that larger myo-inositol dosages may hasten TSH normalization and antibody decrease [45,47,48,49]. The length of the interventions has varied from three to twelve months, as have the requirements for patient inclusion (e.g., baseline TSH cut-offs, antibody thresholds, and pregnancy status). The variation in reported efficacy may be caused by these methodological variations, which also make direct comparisons challenging.

Although the focus of this review is to examine the impact of selenium and myo-inositol in the context of thyroid disease, there is limited evidence to support the use of myo-inositol alone in the treatment of thyroid disease. Most studies investigating the use of myo-inositol in thyroid disease have been conducted in combination with selenium (Table 2), and it is difficult to determine the specific contribution of myo-inositol to the observed effects. Therefore, more research is needed to determine the specific effects of myo-inositol alone in the treatment of thyroid disease. While myo-inositol may have potential therapeutic benefits, the current evidence suggests that it is most effective when used in combination with other supplements such as selenium.

Although the existing evidence is encouraging, it is essential to conduct further well-designed randomized controlled trials to establish conclusive recommendations regarding the use of selenium and myo-inositol supplementation in treating thyroid diseases. Additionally, the influence of factors such as race, ethnicity, age, and sex should be considered when investigating these supplements’ effects on thyroid-related conditions. Future studies should delve into the potential long-term advantages and safety aspects of selenium and myo-inositol supplementation.

Utilizing selenium and myo-inositol supplements as supplementary treatments shows promise in managing thyroid diseases. However, comprehensive research is still necessary to fully comprehend their mechanisms of action and overall clinical importance. Healthcare providers should adopt personalized strategies and refer to the latest evidence when prescribing these supplements to patients with thyroid conditions.

#### 2.1.3. Evidence That Is Neutral or Mixed

Several randomized trials and systematic reviews have reported neutral or inconsistent findings that should be carefully considered, even though many studies report advantages. Randomized trials in Graves’ disease have shown slight biochemical increases in thyroid hormone levels, but no discernible decrease in relapse rates or long-term clinical benefit. According to meta-analyses, the overall effect is still diverse, with results differing depending on the patient demographics and study methodology.

Some interventional trials found that TPOAb and TgAb titers significantly decreased in Hashimoto’s thyroiditis, while TSH levels improved as well. Other studies, however, discovered attenuated or null effects, especially in populations that were selenium-replete or when supplementation was administered at lower dosages and for shorter periods of time. Reviews have underlined that there is still no evidence connecting antibody alterations to long-term clinical outcomes such as better thyroid function or a slower progression to overt hypothyroidism, meaning that the certainty of the evidence is still low to moderate.

These results collectively demonstrate that the advantages of supplements are not universal. Baseline selenium status, iodine intake, regional dietary variations, disease stage, and formulation or dosage selection can all account for variability. Larger, longer, and better-controlled trials that stratify by baseline micronutrient status and use consistent dosages and durations are required in light of these ambiguous and contradictory findings.

#### 2.1.4. Proposed Mechanisms and Modifiers (Se, MYO, Iodine, and Tumor Biology)

Selenium and myo-inositol together may enhance thyroid function through complementary processes. The inositol phosphate–calcium (IP_3_/Ca^2+^) pathway, which controls intracellular calcium release and promotes thyroid hormone production, is how myo-inositol serves as a second messenger of thyroid-stimulating hormone (TSH) [7,41,45,50]. Selenoproteins, such as iodothyronine deiodinases (DIOs), glutathione peroxidases (GPx), and thioredoxin reductases (Trx), which together lessen oxidative stress and permit appropriate T4-to-T3 conversion, depend on selenium [4,9,19,24]. These pathways may work together to lessen tissue damage caused by the immune system and restore euthyroidism.

Additionally, preclinical and early clinical findings point to an involvement in thyroid cancers and nodules. MYO+Se may indirectly reduce proliferative signals by reducing TSH and reducing oxidative stress. While myo-inositol interacts with the PI3K/AKT pathway, which is linked to thyroid carcinogenesis, seleno-enzymes facilitate selenium to prevent the buildup of hydrogen peroxide in the body [23,51]. Even though these results are intriguing, they are still preliminary, and more translational study is necessary before firm conclusions can be drawn.

Myo-inositol and selenium deficits at baseline seem to be significant determinants of therapy response. While insufficient inositol metabolism may lower TSH signal transduction effectiveness [7], selenium shortage has been associated with decreased selenoprotein activity, increased oxidative stress, and an increased risk of autoimmune thyroiditis [2,18,36,47]. Therefore, supplementation may be more beneficial for patients with lower baseline micronutrient levels.

Finally, consideration should be given to the interaction with iodine. Goiter and nodular thyroid disease are caused by iodine shortage, whereas autoimmune thyroiditis can be made worse by excessive iodine intake. Myo-inositol may improve TSH signaling in situations where iodine supply fluctuates, while selenium deficiency may lessen the oxidative damage brought on by excessive iodine exposure [29,44]. Therefore, the effects of supplementation should always be interpreted in light of dietary consumption, geographic location, and baseline iodine status.

Furthermore, geographic location, baseline selenium levels, habitual iodine intake, and disease stage may all affect an individual’s reaction to supplementation [2,29,33,35,39]. Future trial designs should systematically take these things into account.

Safety must also be considered. The therapeutic window for selenium is limited, and long-term consumption of more than 400 µg/day can cause selenosis, which manifests as gastrointestinal issues, brittle nails and hair, dermatological changes, and infrequently, neurological problems [19]. Most studies were brief and used dosages within the upper consumption limits, even though no significant adverse effects were documented in the supplementation trials that were part of this analysis. Though moderate gastrointestinal side effects, e.g., nausea or diarrhea, may occur at greater doses, myo-inositol generally has a favorable safety profile, with daily intakes up to 12 g being regarded as well-tolerated [42]. Crucially, there is currently little information on the long-term safety of myo-inositol and selenium in thyroid populations, highlighting the necessity of organized monitoring in upcoming clinical trials.

## 3. Conclusions

This narrative review examines the role of selenium and myo-inositol supplementation in thyroid diseases, with particular emphasis on hyperthyroidism and hypothyroidism associated with Hashimoto’s thyroiditis and Graves’ disease. Given the thyroid gland’s central role in endocrine regulation, exploring potential adjuvant therapies to mitigate thyroid dysfunction and improve patient outcomes is of considerable clinical importance.

In Hashimoto’s thyroiditis, selenium has been linked to decreases in anti-TPO and anti-Tg antibodies as well as slight improvements in thyroid structure and function [37,41,50]. Additionally, current data indicate that selenium supplementation may help restore biochemical euthyroidism in people with Graves’ disease by lowering levels of thyroid-stimulating receptor antibodies (TRAb) [25]. The positive benefits of selenium are amplified when myo-inositol is added, and it has the potential to improve TSH regulation and lower inflammatory activity in autoimmune thyroid diseases [41,43]. Myo-inositol may decrease nodule size and elasticity ratings for benign thyroid nodules, on the basis of initial research; regardless, confirmatory randomized trials are required [52].

However, the evidence is inconsistent, with some trials indicating attenuated or neutral results [25,35,39,50]. These discrepancies most likely stem from variations in the disease stage, therapy length, dosage and formulation of supplements, geographic iodine availability, and baseline nutritional status.

Personalized supplementing frameworks should be the direction of future strategies. Serum selenium content, selenoprotein P, urine iodine excretion, and antibody titers (anti-TPO, anti-Tg, and TRAb) are potential biomarkers to inform clinical decision-making [18,35,36,47,50]. Some patient subgroups might benefit more than others, including the following:

- Individuals with Hashimoto’s thyroiditis who continue to have positive antibodies despite steady thyroid hormone replacement [41,50];
- Low selenium status and elevated TRAb titers in patients with Graves’ disease [25];
- Postpartum thyroiditis in women, for which selenium has demonstrated protective effects [30,33];
- People who live in areas with low levels of selenium or iodine, where a baseline shortage increases response because resolving baseline deficits can enhance antioxidant selenium enzyme activity (selenium), restore thyroid hormone synthesis and metabolism (iodine), and possibly lower thyroid autoimmunity—as a result, effect sizes may seem larger in deficient settings than in replete populations [2,18,36,47];
- Early research indicates that patients with nodular thyroid disease or subclinical hypothyroidism have improved structurally and functionally [39,43,45,52].

In conclusion, myo-inositol and selenium supplements are promising adjuncts in thyroid care; nonetheless, their best application will rely on baseline micronutrient assessment, cautious patient selection, and consistent dosage schedules. Larger, longer-term randomized controlled trials in a variety of demographics are required to make solid recommendations and elucidate the effectiveness and safety of customized supplementation plans.

## Figures and Tables

**Table 1:** **The use of Selenium in different thyroid diseases**. TPOAb, Thyroid peroxidase antibodies. TPO, Thyroid peroxidase. TgAb, Thyroglobulin antibodies.

Study	Type of Study	Treatment	Inclusion Criteria	Results
Calissendorff et al. (2015) [14]	Interventional study	200 μg/d of selenium vs. placebo for 36 weeks	38 patients with untreated thyrotoxicosis	FT4 decreased at 18 weeks (*p* = 0.01) and also at 36 weeks (*p* = 0.01) in the Se group. The TSH increased more in the Se group at 18 weeks (*p* = 0.04).
Jadwiga Kryczyk-Koziol et al. (2021) [38]	Interventional study	Sodium selenite (IV) at a dose of 100 µg/day for 6 months	36 newly diagnosed Hashimoto’s thyroiditis patients with euthyroidism or subclinical hypothyroidism	Restricted the progress of overt hypothyroidism and also decreased levels of TPO (*p* = 0.02).
Lan-Feng Wang et al. (2021) [34]	Interventional study	Selenium yeast oral supplementation of 200 µg/day for 15 months	100 patients with autoimmune thyroiditis	TGAb titers and TPOAb markedly decreased compared to baseline (*p* < 0.05).
Esther J. van Zuuren et al. (2014) [35]	Cochrane review	Selenium supplementation (83–200 µg/day; 3–12 months)	463 participants with Hashimoto’s thyroiditis	Selenium significantly reduced antibody levels (*p* <0.001).
Pirola et al. (2020) [39]	Interventional study	83 µg/day selenium supplementation for 6 months	50 subclinical hypothyroidism patients due to Hashimoto’s thyroiditis	TSH level maintained for the entire study duration for the responders vs. non-responders (42.6% vs. 6.8%; *p* < 0.001).
G. Mantovani et al. (2019) [30]	Randomized controlled study (RCT)	83 µg/day selenium supplementation vs. placebo during pregnancy and for 6 months after delivery	45 pregnant women with thyroiditis	Depletion of autoantibodies during pregnancy and postpartum thyroid recurrence (*p* < 0.01).
Mazokopakis et al. (2007) [32]	Interventional study	200 mcg of selenium supplementation for 6 months	80 women with Hashimoto’s thyroiditis	Significant reduction in anti-TPO levels (*p* < 0.0001).

**Table 2:** The use of myo-inositol and Selenium in different thyroid diseases. MYO, Myo-inositol. Se, Selenium. RCT, Randomized Controlled Trial. TSH, Thyroid Stimulating Hormone. Thyroid peroxidase antibodies.

Study	Type of Study	Treatment	Inclusion Criteria	Results
Nordio et al. (2017) [41]	RCT	Myo-inositol, 600 mg/day + selenium 83 µg for 6 months	86 patients with Hashimoto’s disease and subclinical hypothyroidism	Restoring a normal thyroid function by improvement in TSH levels and improvement in the quality of life
G. Porcaro et al. (2018) [40]	RCT	MYO (600 mg/day) + Se (83 µg/day) supplementation vs. no treatment	Pregnant women (1st to the 3rd trimester)	Normalization of TSH, FT3 and FT4
Nordio, M et al. (2013) [43]	RCT	MYO (600 mg/day) + Se (83 µg/day) supplementation vs. 83 µg Selenium	Autoimmune thyroiditis patients and TPOAb > 350 IU/mL, TSH (4.01–9.99 mIU/L,) and a normal FT4 level	Myo-inositol treatment lowers TSH levels directly, due to its action as a TSH second messenger *p* < 0.01

## Data Availability

No new data were created or analyzed in this study. Data sharing is not applicable to this article.

## Abbreviations

**Abbreviation**	**Full Form**
AITD	Autoimmune Thyroid Disorders
CD4	Cluster of Differentiation 4
FT3	Free Triiodothyronine
FT4	Free Thyroxine
GD	Graves’ Disease
GO	Graves’ Orbitopathy
GPx	Glutathione Peroxidase
H_2_O_2_	Hydrogen Peroxide
HPT axis	Hypothalamic–Pituitary–Thyroid axis
HT	Hashimoto’s Thyroiditis
IL	Interleukins
IRB	Institutional Review Board
MYO	Myo-Inositol
PBMCs	Peripheral Blood Mononuclear Cells
PPT	Postpartum Thyroiditis
PTM	Pretibial Myxedema
RCT	Randomized Controlled Trial
Se	Selenium
T3	Triiodothyronine
T4	Thyroxine
TgAb	Thyroglobulin Antibody
TNF	Tumor Necrosis Factor
TPO	Thyroid Peroxidase
TPOAb	Thyroid Peroxidase Antibodies
TRAb	TSH Receptor Antibodies
TRH	Thyrotropin-Releasing Hormone
TRx	Thioredoxin Reductase
TSH	Thyroid-Stimulating Hormone
TSHR	Thyroid-Stimulating Hormone Receptor
